# DNA Demethylation and USF Regulate the Meiosis-Specific Expression of the Mouse *Miwi*

**DOI:** 10.1371/journal.pgen.1002716

**Published:** 2012-05-17

**Authors:** Yu Hou, Jia Yuan, Xiang Zhou, Xiazhou Fu, Hanhua Cheng, Rongjia Zhou

**Affiliations:** Department of Genetics, College of Life Sciences, Wuhan University, Wuhan, China; Stanford University School of Medicine, United States of America

## Abstract

Miwi, a member of the Argonaute family, is required for initiating spermiogenesis; however, the mechanisms that regulate the expression of the *Miwi* gene remain unknown. By mutation analysis and transgenic models, we identified a 303 bp proximal promoter region of the mouse *Miwi* gene, which controls specific expression from midpachytene spermatocytes to round spermatids during meiosis. We characterized the binding sites of transcription factors NF-Y (Nuclear Factor Y) and USF (Upstream Stimulatory Factor) within the core promoter and found that both factors specifically bind to and activate the *Miwi* promoter. Methylation profiling of three CpG islands within the proximal promoter reveals a markedly inverse correlation between the methylation status of the CpG islands and germ cell type–specific expression of *Miwi*. CpG methylation at the USF–binding site within the E2 box in the promoter inhibits the binding of USF. Transgenic Miwi-EGFP and endogenous Miwi reveal a subcellular co-localization pattern in the germ cells of the *Miwi-EGFP* transgenic mouse. Furthermore, the DNA methylation profile of the *Miwi* promoter–driven transgene is consistent with that of the endogenous *Miwi* promoter, indicating that *Miwi* transgene is epigenetically modified through methylation *in vivo* to ensure its spatio-temporal expression. Our findings suggest that USF controls *Miwi* expression from midpachytene spermatocytes to round spermatids through methylation-mediated regulation. This work identifies an epigenetic regulation mechanism for the spatio-temporal expression of mouse *Miwi* during spermatogenesis.

## Author Summary

Germ cell differentiation is a key process in the formation of functional spermatozoa. Despite the wealth of information about gene expression patterns and regulations important for this process, it is not clear how spatio-temporal expression of the key factor *Miwi* during spermatogenesis is controlled. We have characterized the functional promoter of the mouse *Miwi* gene. Transgenic mice harboring *EGFP* under the *Miwi* core promoter containing just the functional CCAAT box and E2 box were generated and demonstrated that it can direct germ cell–specific expression. We further identified the transcription factors NF-Y and USF1/2 as activators of *Miwi* gene expression, through their binding to the CCAAT box and E-box/E2 site of the *Miwi* promoter, respectively. A CpG dinucleotide just located within the USF binding site is responsible for mediating methylation-dependent silencing of the *Miwi* gene. Our findings provide new insight into an epigenetic regulation mechanism for the spatio-temporal expression of the mouse *Miwi* during spermatogenesis.

## Introduction

Spermatogenesis is a complex process consisting of two types of cell division, mitosis and meiosis. Meiosis is initiated in B spermatogonia, eventually leading to the production of spermatozoa. Spermatozoa generated in the testis enter the epididymis and undergo maturation processes necessary for them to gain motility and become capable of fertilization. During spermatogenesis, differential gene expression is orderly and accurately regulated at the transcriptional level to ensure differentiation of germ cells [1]. Male germ cells in the adult mouse exhibit a highly distinct methylation pattern, and both *de novo* methylation and demethylation occur during spermatogenesis [2], [3]. DNA methylation of postmigratory germ cell-specific genes is also observed in both premigratory germ cells and somatic cells [4]. These methylation modifications in germ cells are important for accurate spermatogenesis. Another salient feature of gene expression regulation during spermatogenesis is translational delay or repression [5], [6], [7]. After transcription, some mRNAs are assembled in ribonucleoprotein particles, which contain RNA-binding proteins, export proteins and processing factors. Transcripts are then transported through nuclear pore complexes into the cytoplasm, where they join in formation of the chromatoid body (CB) for both storage and processing after meiosis [8]. Evidence is accumulating that microRNAs (miRNAs) can also reduce translation [9]. miRNAs likely mediate translational repression by decreasing the rate of translation initiation.

Miwi, a PIWI subfamily member of Argonaute protein family, is characterized by conserved PAZ and Piwi domains for RNA-binding, and required for initiating spermiogenesis [10]. In mice, there are three Piwi members (Miwi, Mili/Piwil2 and Miwi2/Piwil4). Miwi interacts directly with piwi-interacting RNAs (piRNAs) [11], [12], [13], and is required for the expression of not only piRNAs but also a subset of miRNAs [14]. Miwi is a cytoplasmic protein expressed specifically in the testis from midpachytene spermatocytes to round spermatids [10], [15]. Particularly, Miwi is present throughout the cytoplasm in spermatocytes, and gradually concentrates in the CB in round spermatids [14], [16]. A complex formation between Miwi, Mili and Tdrd1 is critical for the integrated subcellular localizations of these proteins [17]. The Piwi interactome has revealed that arginine methylated Miwi can interact with multiple tudor domain containing proteins [18], [19]. Tudor proteins recognize methyl-arginine of Piwi proteins, driving the localization of Piwi proteins to the CB [19]. It has been suggested that Tudor proteins regulate biological functions of Piwi proteins via specific association with methylation modifications of the Piwi arginine [20]. Despite the wealth of information, expression regulation mechanisms of the gene *Miwi* remain poorly understood.

In this study, we have identified the functional promoter of the mouse *Miwi*. *Miwi-EGFP* transgenic mice reveal that a 303 bp core promoter of the mouse *Miwi* gene directs specific expression during meiosis. Notably, transcription factors NF-Y (Nuclear Factor-Y) and USFs (Upstream Stimulatory Factors) bind to and activate the promoter of *Miwi*. Methylation analysis of the CpG islands in the *Miwi* promoter reveals that CpG methylation at the USF-binding site within the E2 box inhibits the binding of USFs. In addition, both transgenic *Miwi-EGFP* and endogenous *Miwi* reveal a co-localization pattern in *Miwi-EGFP* transgenic mouse testis. Furthermore, the promoter region of *Miwi*-*EGFP* transgene in transgenic mice is epigenetically modified *in vivo* as that of the endogenous *Miwi*. These results suggest an epigenetic regulation mechanism for the spatio-temporal expression of the mouse *Miwi* during spermatogenesis.

## Results

### Identification of the mouse *Miwi* promoter

**Figure 1:**
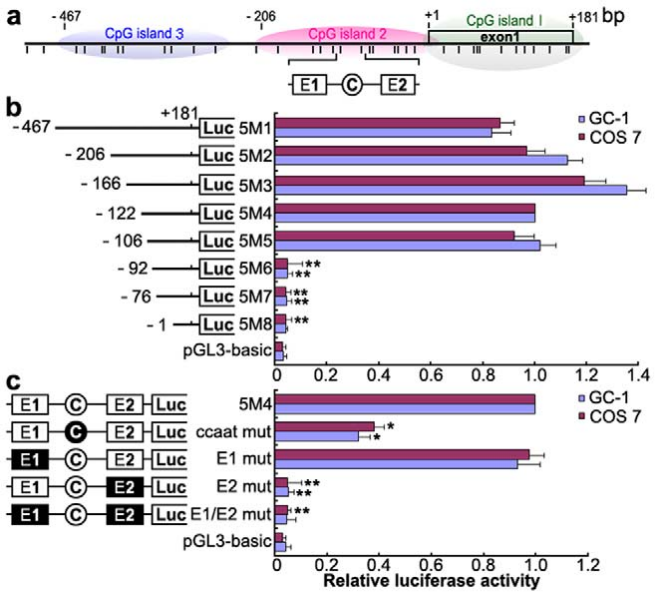
Deletion and point mutation analysis of the *Miwi* promoter. a), Schematic diagram of the CpG islands and E1, E2 and CCAAT boxes in the *Miwi* promoter. The short vertical bars represent the CpG dinucleotides. b), Promoter activities of a series of deleted constructs determined by luciferase assay. Left panel, schematic representation of the mutants linked with luciferase gene in pGL3 vector. The nucleotides are numbered from the potential transcriptional start site that was assigned +1. Right panel, the relative activities to the 5M4 construct of a series of mutant constructs determined by luciferase assays. c), Point mutation analysis in the E1, E2 and CCAAT boxes of the *Miwi* promoter by luciferase assay. The 5M4 construct of the promoter (303 bp) was used for these point mutation analyses by linkage to the luciferase gene. The relative activities to the 5M4 construct of the mutant constructs were determined by luciferase assays. The open box and open circle indicate the intact binding site of E1 and E2 or CCAAT box respectively. The filled box or circle indicates the corresponding mutations. Non modified pGL3 was used as a negative control. The firefly luciferase activity was normalized to the Renilla luciferase activity, and the data is shown as the fold increase/decrease over the luciferase activity of pGL3-5M4. The results are the mean ± S.D. of three independent experiments. *, P<0.05; **, P<0.001 compared to the 5M4 wild-type construct.

To identify the promoter region and regulatory elements of mouse *Miwi*, a series of deletions of the potential promoter were introduced upstream of the luciferase gene based on the prediction of CpG islands. Luciferase activity was determined in both GC-1 and COS7 cells. Promoter analysis has revealed that the 5′ flanking sequence from −106 to −92 is important for its transcriptional activity (Figure 1a, 1b). Because the *Miwi* gene lacks a TATA box, we identified putative transcription elements using the TFSEARCH software (http://www.cbrc.jp/research/db/TFSEARCH.html). A CCAAT box (−97∼−93) and two E boxes (−107∼−102, −82∼−77) were observed within the core promoter region from −106 to −92, which are putative binding sites of transcription factors NF-Y and USF respectively. To functionally determine the importance of these elements, site-directed mutagenesis was performed using wild type pGL3-5M4 construct as a template. The mutated CCAAT box or E2 box showed obvious decreases in promoter activity, as compared to the wild type pGL3-5M4 construct, especially the E2 mutant, where very little transcription was observed (Figure 1c). Meanwhile, mutations for E1 box had no effect on transcription activity. The results indicate that both the CCAAT box and the E2 boxes are important for *Miwi* promoter activity.

### Transcription factors NF-Y and USF bind to the *Miwi* promoter both *in vitro* and *in vivo*

**Figure 2:**
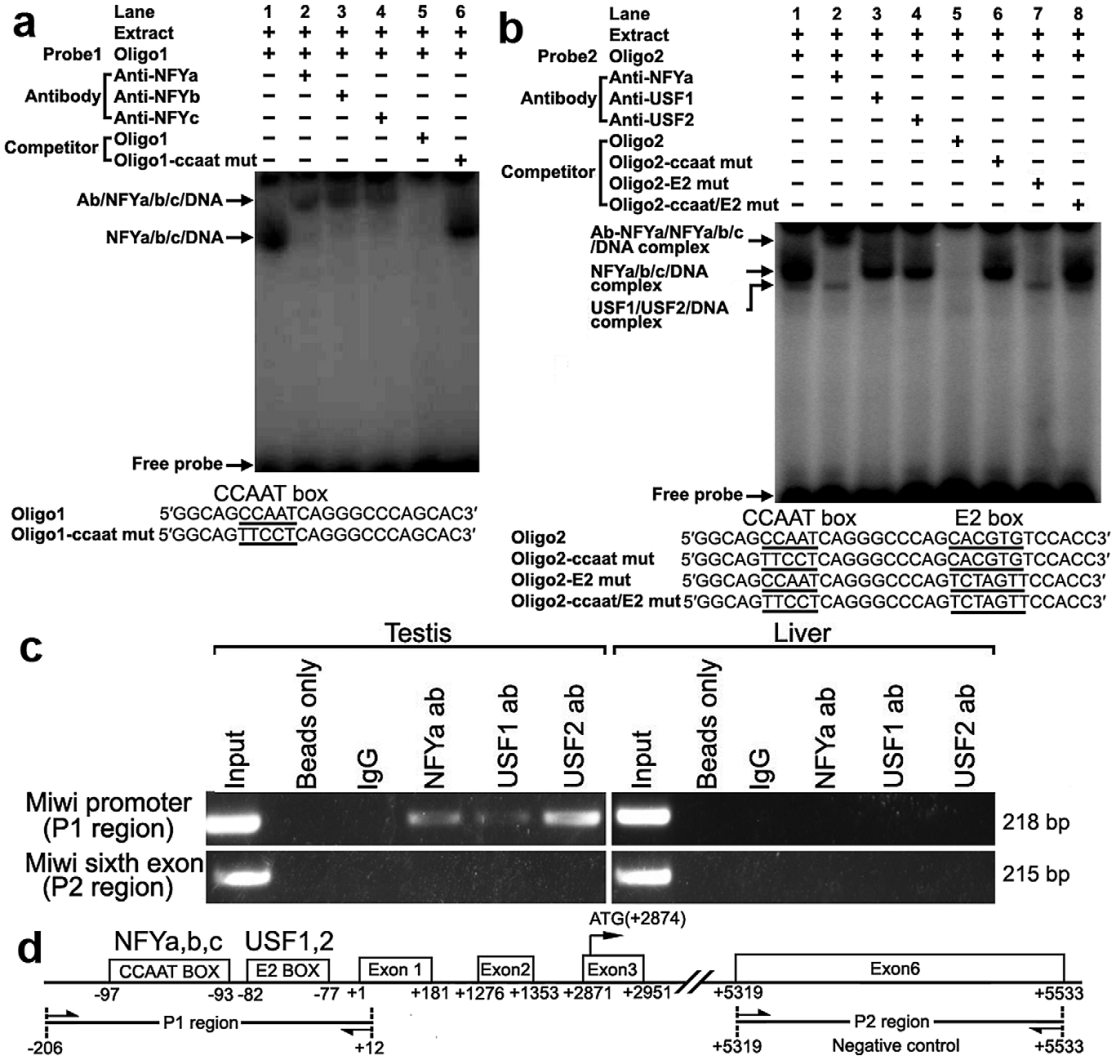
Electrophoretic mobility shift and ChIP assays of NF-Y and USF binding to the *Miwi* promoter. a), NF-Y binding to *Miwi* promoter. The oligo1 corresponding to −102/−80 was γ-^32^P-ATP labeled and incubated with 5 µg of nuclear extract of mouse testis in the absence or presence of 50-fold excess of various competitor DNA oligos (mutant or non-labeled oligo1) or antibodies (NF-Ya, NF-Yb and NF-Yc), as indicated on the top of the gel image. The specific DNA/protein complex and the super-shift bands are indicated by arrows. The DNA/protein complex formation cannot be affected by adding competitor mutated DNA (lane 6). The sequences of oligo1 and corresponding mutant are show under the panel. b), USF binding to *Miwi* promoter. The oligo2 corresponding to −102/−71 was γ-^32^P-ATP labeled and incubated with 5 µg of nuclear extract of mouse testis in the absence or presence of 50-fold excess of various competitor DNA oligos (mutants or non-labeled oligo2) or antibodies (NF-Ya,USF1/2) as indicated. The specific DNA/protein complex and the super-shift band are indicated by arrows. The sequence of oligo2 and corresponding mutants are indicated under the panel. Two distinct migrating complexes were detected (lane 1). Supershift complex with anti-NFYa showed in lane 2. The addition of anti-USF1 or anti-USF2 antibody to the binding reaction caused the disappearance of the DNA/protein complex (lane 3,4). The DNA/protein complex formation can not be affected by adding mutated DNA competitor (lane 6,7,8). c), ChIP assay of NF-Y and USF binding to the *Miwi* promoter in testis. The interaction of NF-Y and USF *in vivo* with the *Miwi* promoter was determined by chromatin immunoprecipitation analysis. Mouse testis and livers were chopped into small pieces in 1% formaldehyde to cross-link endogenous proteins and DNA. Samples of sonicated chromatin were immunoprecipitated with anti-NFYA, anti-USF1, anti-USF2, no antibody (beads only) or preimmuno IgG (control) respectively. DNA isolated from immunoprecipitated material was amplified by PCR with primers to amplify the 218-bp mouse *Miwi* promoter sequence corresponding to the −206 to +12 region. Primers for an unrelated part of the sixth exon of *Miwi* were used as negative controls. The amplified PCR fragments were analyzed on 2% agarose gel. d), The relative positions of the primers in the ChIP assay.

To determine binding of transcription factors NF-Y and USF to the CCAAT and E2 boxes of the promoter respectively, electrophoretic mobility shift assays were carried out using nuclear extracts prepared from mouse testis and double stranded oligonucleotides. Incubation of a CCAAT probe spanning −102/−80 with nuclear extract gave rise to the formation of a DNA–protein complex. The addition of an excessive amount of unlabelled oligo DNA, but not the mutated CCAAT box, could compete with this binding (Figure 2a). Furthermore, addition of anti-NFYa, anti-NFYb or anti-NFYc antibody to the binding reaction caused the disappearance of the specific DNA/protein complex, and the appearance of the super-shift band (Figure 2a). These results show that the CCAAT box located from −97/−93 of the *Miwi* promoter region is capable of binding with transcription factors NF-Ya, NF-Yb and NF-Yc *in vitro*.

With use of nuclear extracts prepared from mouse testis and oligo spanning −102/−71 bearing the CCAAT and E2 boxes, two specific DNA/protein complexes were observed (Figure 2b). Both of the complexes could be competed by the addition of a 50-fold molar excess of unlabelled oligo DNA, but not by the mutated CCAAT box or E2 box, or mutant of both sites. Furthermore, the addition of anti-NF-Ya antibody to the binding reaction generated a super-shift band, and the addition of anti-USF1 or anti-USF2 antibody caused the disappearance of the DNA/protein complex (Figure 2b). These results indicate that both transcription factors USF1 and USF2 bind to the E2 box located from −82/−77 of the *Miwi* promoter region *in vitro*.

To determine whether NF-Y and USF bind to the mouse *Miwi* promoter *in vivo*, we performed chromatin immunoprecipitation (ChIP) analysis. As shown in Figure 2c, a 218 bp DNA fragment was amplified from the precipitates of anti-NF-Ya, anti-USF1 and anti-USF2 in testis, but it was not amplified from liver (control tissue)(Figure 2c). The amplified fragments were confirmed by sequencing. To further rule out the possibility that the *Miwi* promoter region precipitated by anti-NF-Y, anti-USF1 and anti-USF2 is due to non-specific binding of these antibodies, an additional PCR amplification of a distinct genomic region (exon 6) was performed on all of the precipitated chromatin DNAs. No band from the precipitate by anti-NFY, anti-USF1 or anti-USF2 antibody was observed. These results indicate that NF-Y, USF1 and USF2 specifically bind to the *Miwi* promoter region *in vivo*.

### NF-Y and USF activate the *Miwi* promoter

**Figure 3:**
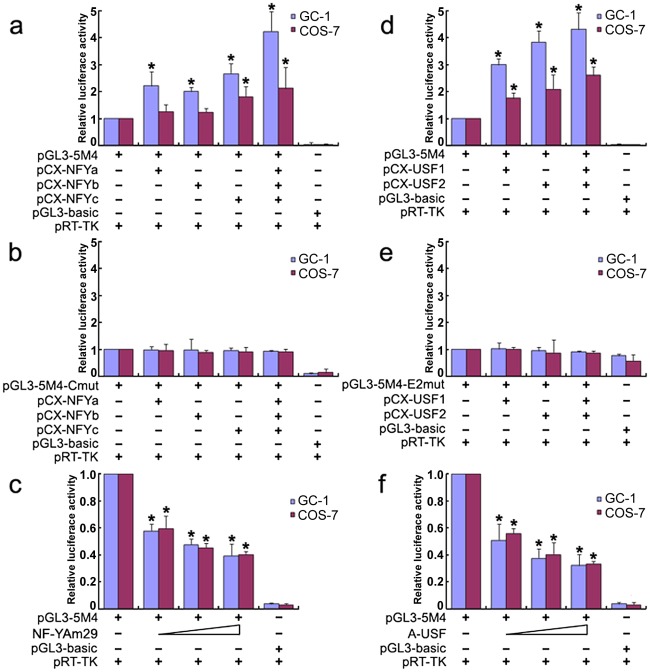
Over-expression of NF-Y or USF increases *Miwi*-luciferase activity. a), Over-expression of NF-Y upregulates *Miwi*-luciferase activity. GC-1 or COS7 cells were transfected with 0.2 µg pGL3-5M4 and 0.2 µg NF-Y expression plasmids (pCX-NFYa, pCX-NFYb, pCX-NFYc) as indicated. b), The mutated CCAAT box construct pGL3-5M4-Cmut (0.2 µg) contransfected with NF-Y expression plasmids (pCX-NFYa, pCX-NFYb, pCX-NFYc) as indicated, does not increase the *Miwi*-luciferase activity. c), The dominant negative NF-Ya expression plasmid NF-YAM29 (0.1 µg, 0.2 µg, 0.3 µg) was cotransfected with 0.2 µg pGL3-5M4, which inhibits *Miwi*-luciferase activity. d), Over-expression of USF1/2 upregulates *Miwi*-luciferase activity. pGL3-5M4 (0.2 µg) was cotransfected with 0.2 µg expression plasmids (pCX-USF1 and/or pCX-USF2) into GC-1 or COS7 cells. e), E2 box mutant pGL3-5M4-E2mut (0.2 µg) was cotransfected with 0.2 µg USF1 and/or USF2 expression plasmids (pCX-USF1 and/or pCX-USF2), indicating no increase in luciferase activity. f), The dominant negative USF expression plasmid A-USF (0.1 µg, 0.2 µg, 0.3 µg) was cotransfected with 0.2 µg pGL3-5M4, which inhibits *Miwi*-luciferase activity. pGL3-basic was employed as a negative control. pRT-TK served as an inner control for transfection efficiency. The relative luciferase activities to the 5M4 construct were determined by luciferase assay. Cells were harvested 24 hours post-transfection, and luciferase activity was measured and normalized to Renilla luciferase activity. All transfection experiments were repeated at least three times and the data are shown as the fold increase or decrease over the luciferase activity of pGL3-5M4. Mean ± S.D. are shown. *, P<0.05, as compared to the 5M4 wild-type construct.

To further investigate the role of transcription factors NF-Y and USF in activation of the *Miwi* promoter, both GC-1 and COS7 cells were co-transfected with luciferase reporter driven by *Miwi* promoter or the mutant for CCAAT or E2 sites with expression plasmid for NF-Ya/b/c or USF1/2 respectively. The luciferase activity increased when co-transfected with NF-Ya/b/c or USF1/2 (Figure 3a, 3d). However, the activities in the mutants for CCAAT or E2 sites were not upregulated (Figure 3b, 3e). Replacing the NF-Ya/b/c or USF1/2 expression plasmid with relevant dominant-negative constructs (NF-YAm29 and A-USF) resulted in a clear reduction of luciferase activity in a dosage-dependent manner (Figure 3c, 3f). NF-YAm29 has a three amino acid substitutions in the C-terminal region of the NF-Ya, which impairs DNA binding activity and acts as a dominant-negative mutant by sequestering the NF-Yb/Yc subunits into a defective complex [21]. A-USF contains the USF heterodimerization domain but lacks the USF-specific region, which is required for transcriptional activation [22], [23]. The results suggest that NF-Y and USF activate the *Miwi* promoter.

### Methylation profile of the CpG islands in the *Miwi* promoter

**Figure 4:**
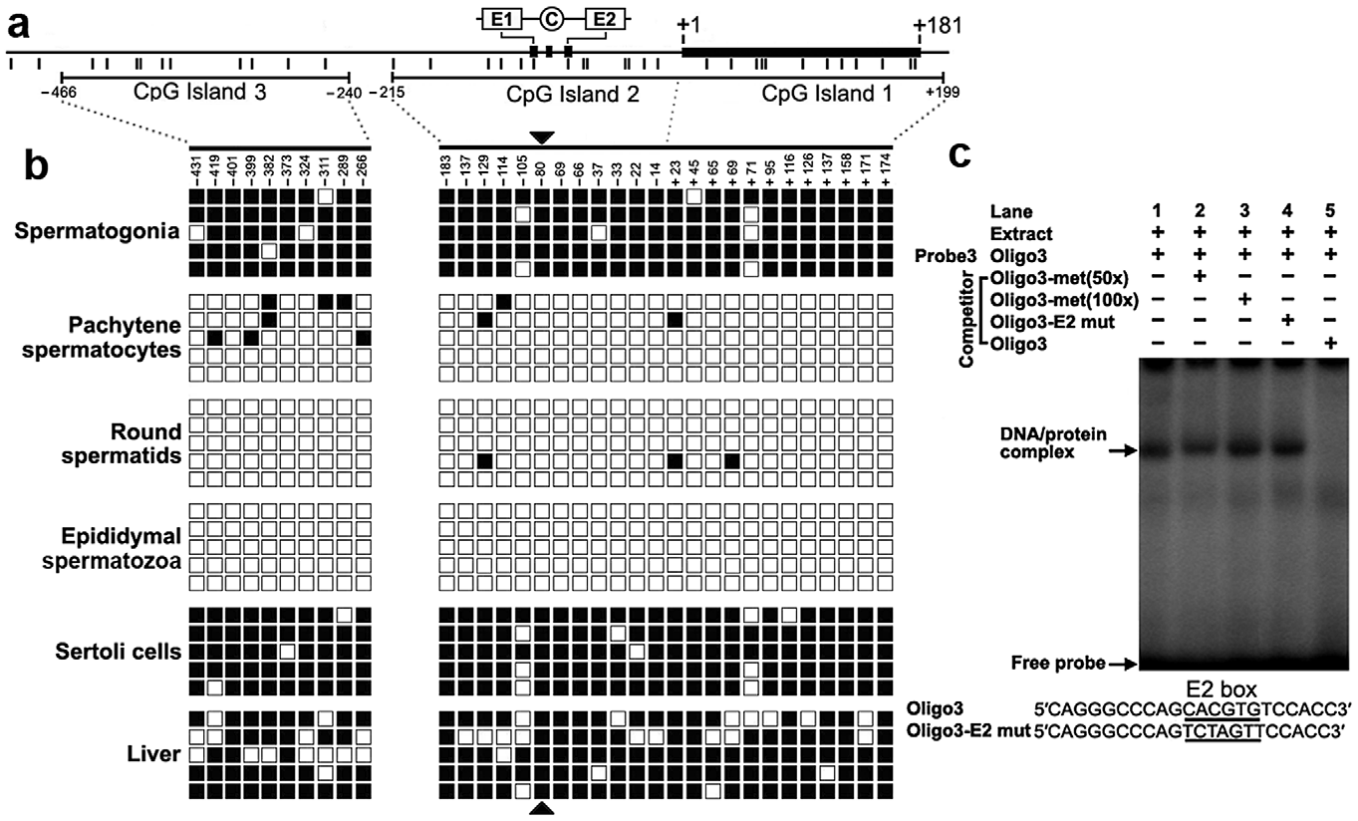
The cytosine methylation profile of the CpG islands in the *Miwi* promoter, and methylation at the E2 box inhibits USF binding. a), Schematic depiction of the CpG islands and CpG dinucleotides (short verticals) spanning the promoter. The relative positions of the E1, E2 and CCAAT box are indicated. b), Methylation status of each CpG dinucleotide in three CpG islands in various cell types of testis and liver. The spermatogonia, pachytene spermatocytes, round spermatids, epididymal spermatozoa and Sertoli cells were isolated from adult testis by flow cytometry. Bisulfite-treated genomic DNA was amplified and cloned into T-easy vector. Five independent clones from each sample were sequenced. Each row represents the sequence of an individual clone, whereas, each column depicts the position of each CpG site relative to the potential transcription start site. Open squares indicate unmethylated CpG sites; filled squares indicate methylated CpG sites; black triangles represent CpG site in the E2 box. c), The CpG methylation at the E2 box inhibits USF binding. The oligo3 corresponding to −92/−71 was γ-^32^P-ATP labeled and incubated with 5 µg of nuclear extract prepared from mouse testis in the absence or presence of various competitor DNA, as indicated on the top of the gel image. Nuclear extracts were incubated with labeled oligo3 in the absence (lane 1) or in the presence of various DNA competitors: 50-fold or 100-fold excess methylated oligo3 (lanes 2,3), mutant oligo3 (lane 4) or non-labeled oligo3 (lane 5). The specific DNA/protein complexes were indicated by arrows. The sequences of oligo3 and mutant competitor are shown under the panel.

We detected three CpG islands that span positions −466 to −240, −215 to +17, and −7 to +199 around the 2 kb promoter region using the MethPrimer program (Figure 4a). To determine the methylation status of the three CpGs islands, we performed bisulfite sequencing analysis using DNA isolated from spermatogonia, pachytene spermatocytes, round spermatids, epididymal spermatozoa, Sertoli cells and liver tissue. The CpG islands from bisulfite-treated DNA were amplified and five randomly selected clones were subjected to sequencing. Methylation analysis of the three CpG islands in the *Miwi* promoter revealed a differential methylation profile of CpGs among these cell types (Figure 4b). The CpG islands were always hypermethylated in the spermatogonia, Sertoli cells and liver tissue where *Miwi* was not expressed, while the CpG islands were unmethylated in the pachytene spermatocytes and round spermatids where *Miwi* was expressed. The CpG islands were also unmethylated in the epididymal spermatozoa, where *Miwi* was not expressed.

### CpG methylation at the USF–binding site within the E2 box inhibits the binding of USF

From the methylation profile of CpG islands in the *Miwi* promoter, we noted an inverse association between the methylation status of the CpG dinucleotide (−80) within the E2 box and the *Miwi* expression pattern. To examine the importance of CpG methylation within the E2 box for *Miwi* expression, we performed EMSAs using a methylated double-stranded oligonucleotide, in which the cytosine residue in the CpG dinucleotide was methylated with M.SssI (CpG Methyltransferase) *in vitro*. Incubation of nuclear extract from mouse testis with a wild-type oligo probe resulted in the formation of a protein/DNA complex. This complex could be competed with an excessive amount of unlabelled oligo DNA. However, the addition of 50-excess or 100-excess of unlabelled methylated oligo DNA or E2 mutant did not affect the complex formation (Figure 4c). Our previous ChIP experiment in testis and liver showed that USF1/2 could bind to the E2 box only in testis (Figure 2), which is consistent with the methylation status at the CpG site within the E2 box. These results suggest that CpG methylation at the USF-binding site within the E2 box inhibits the binding of USF.

### A 303 bp core promoter of the mouse *Miwi* directs specific expression during meiosis in transgenic mice

**Figure 5:**
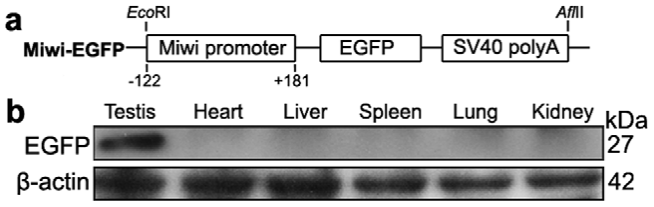
*Miwi*-*EGFP* transgenic construct and expression in transgenic mice. a), Schematic depiction of transgenic *Miwi*-*EGFP* construct, which contains a region of the *Miwi* promoter from −122∼+181 cloned into a pEGFP-1 vector. The *Eco*RI-*Afl*II fragment was used for microinjection. b), Western blot analysis of the EGFP protein in the adult testis, heart, liver, spleen, lung and kidney in *Miwi*-*EGFP* transgenic mouse. The EGFP expression was only detected in the testis. β-actin served as an internal control.

**Figure 6:**
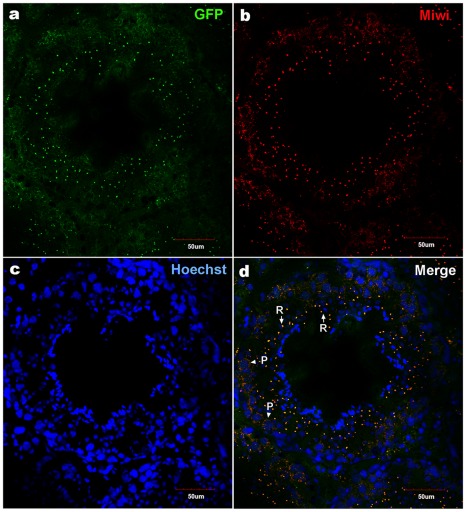
Co-localization of transgenic Miwi-EGFP and endogenous Miwi in transgenic mouse. Transgenic Miwi-EGFP and endogenous Miwi proteins in the *Miwi*-*EGFP* transgenic mouse testis were examined by indirect immunofluorescence and confocal microscopy using anti-GFP and anti-Miwi antibodies. Transgenic *Miwi-EGFP* (a) and endogenous *Miwi* (b) are nearly co-expressed in the cytoplasm of spermatocytes (P, arrowheads in panel d) and round spermatids (R, arrows in panel d), especially in round spermatids, both Miwi and GFP concentrate in the chromatoid body. Nuclei were stained using Hoechst33258 (blue). Green, transgenic EGFP; Red, endogenous Miwi; Merged image was showed in panel d. Negative controls were showed in Figure S1A.

**Figure 7:**
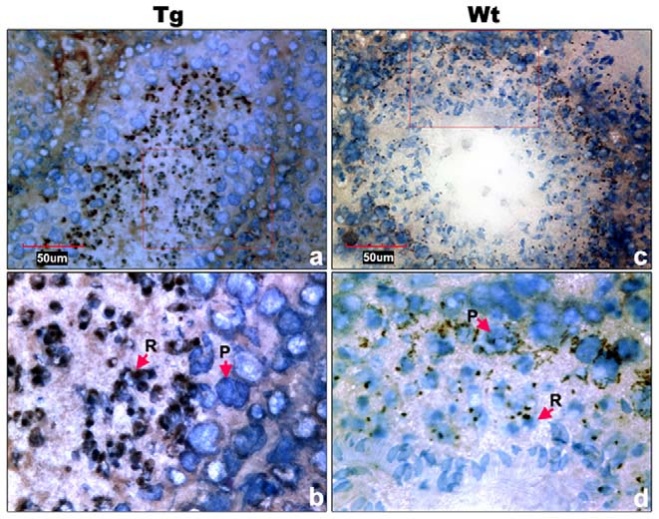
Cell types of Miwi-EGFP expression in transgenic mouse testis. a), Immunocytochemical localization of EGFP in transgenic mouse testis using anti-EGFP antibody. c), Endogenous Miwi expression was detected in the wild-type testis using anti-Miwi antibody. Signals were detected with horseradish peroxidase-conjugated secondary antibody (brown). Bottom panels (b,d) are enlarged images (100×) from the squares in their respective upper panels (a,c) (40×). Negative controls were showed in Figure S1B. Nuclei were re-dyed with hematoxylin (blue). P, pachytene spermatocytes; R, round spermatids.

To examine the functional role of the core promoter of *Miwi* during spermatogenesis, we have made *Miwi*-drived *EGFP* transgenic mice. The *Miwi* promoter sequence spanning from −122 to +181 was cloned into the pEGFP-1 vector. A 1.3 kb fragment digested with both HindIII and AflII was purified for the production of transgenic mice (Figure 5a). Western blot analysis revealed that *Miwi*-drived *EGFP* was specifically expressed in testis of transgenic founders (Figure 5b). To determine whether transgenic *Miwi-EGFP* and endogenous *Miwi* are co-expressed in the seminiferous tubules of transgenic mouse testis, we first examined the subcellular localization by immunofluorescence and confocal microscopy. Transgenic *Miwi-EGFP* and endogenous *Miwi* were nearly co-localized in the cytoplasm of spermatocytes and round spermatids, particularly in round spermatids, both Miwi and EGFP concentrate in the chromatoid body (Figure 6). The cell types expressing EGFP in the seminiferous tubules of transgenic mice were further confirmed by immunochemical localization analysis in both transgenic and wild mice (Figure 7). These results suggest that *EGFP* transgene expression, driven by the 303 bp *Miwi* promoter, is germline-specific during spermatogenesis. Other promoters (e.g. *Dazl* and *H1t*) have also been observed to drive reporter expression in a germ-cell specific manner [24], [25].

### The *Miwi*-*EGFP* transgene promoter is epigenetically modified through methylation *in vivo* in transgenic mice

**Figure 8:**
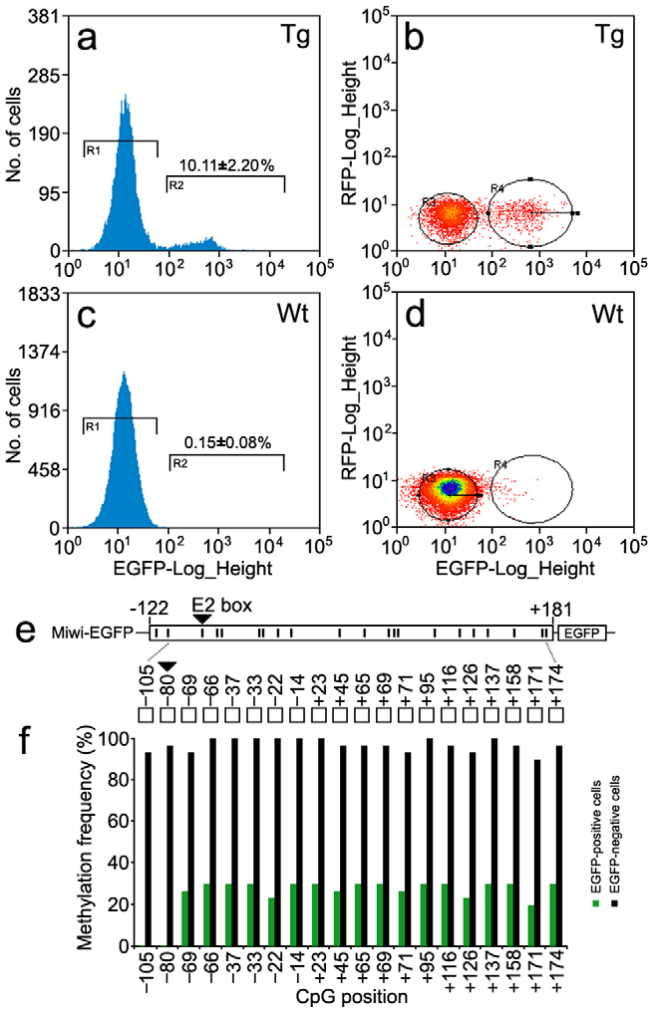
Methylation profile of the *Miwi* promoter–driven transgene in *Miwi*-*EGFP* transgenic mice. a,c), Flow cytometric analysis of male germ cells from heterozygous *Miwi*-*EGFP* transgenic mice (a) and wild type (c). Values on the y-axis represent the number of cells, and values on the x-axis represent EGFP fluorescence intensity. The number in the R2 gate represents the percentage of the cells with EGFP fluorescence. The data is representative of three independent experiments. b,d) Flow cytometric separation and collection of EGFP-positive male germ cells from *Miwi*-*EGFP* transgenic mice (b) and wild type mice were used as a control (d). Values on the x-axis represent EGFP fluorescence intensity, and values on the y-axis represent red fluorescence intensity. The cell population in the R4 gate shows clear EGFP fluorescence in panel b. e). The positions of 20 CpG dinucleotides within the promoter region of the *Miwi*-*EGFP* transgene. f). Comparison of methylation frequencies along the CpG positions between EGFP-positive cells and EGFP-negative cells from the *Miwi*-*EGFP* transgenic mouse testis. The CpG dinucleotides in the E2 box (−80) were hyper-methylated (96%) in the EGFP negative cells, however, the CpG in the same position were completely un-methylated in the EGFP-positive cells.

To verify whether the DNA methylation profile of the *Miwi* promoter driven transgene matches that of the endogenous *Miwi* promoter, we performed flow cytometric analysis of male germ cells from the testis of three *Miwi*-*EGFP* heterozygous transgenic mice. Around 10% of cells were EGFP-positive in the transgenic mice (Figure 8a, 8b), while male germ cells from wild-type mice did not contain EGFP-positive cells (Figure 8c, 8d). To compare the methylation status of both EGFP-positive and negative cells in the transgenic mice testis, both types of cells were separated and collected from three heterozygous transgenic *Miwi*-*EGFP* mice. We then performed bisulfate sequencing analysis using DNA isolated from both EGFP-positive and negative cells. Sixty randomly selected clones were subjected to sequencing. Biased methylation status was observed between EGFP-positive cells and negative cells from the testes of *Miwi*-*EGFP* transgenic mice. Methylation frequency for EGFP-negative cells was 90–100%, however the frequency for EGFP-positive cells was lower than 30%. Further, in the CpG positions −105 and −80 (key USF binding site), the methylation frequency for EGFP-negative cells was 93% and 96% respectively, while the CpG dinucleotides in both positions of the EGFP-positive cells were un-methylated (Figure 8e, 8f). These results show that the DNA methylation profile of the *Miwi* promoter driven transgene is completely consistent with that of the endogenous *Miwi* promoter and further demonstrate that USF and demethylation of its binding site regulate male germline-specific expression of *Miwi* gene.

## Discussion

To produce functional sperm, male germ stem cells gradually lose their stem cell potential and initiate differentiation to become highly specialized spermatozoa. During the differentiation process, accurate spatio-temporal expression regulation of a variety of genes is important to germ cell fate. The current study reveals a clear methylation regulatory mechanism of the *Miwi* gene during spermatogenesis; CpG methylation inhibits the binding of the USF, thereby repressing *Miwi* expression. When demethylation occurs, the transcription factor USF binds to and activates the *Miwi* promoter. Importantly, methylation-mediated regulation of *Miwi* by transcription factor USF occurs in a small region of the *Miwi* core promoter, which controls germline-specific expression of *Miwi* in testis. Thus, we present an epigenetic regulation mechanism for the spatio-temporal expression of mouse *Miwi*, which is driven by transcription factor USF during spermatogenesis.

The present study has contributed to our understanding of how ubiquitous protein USF regulates testis-specific expression of the *Miwi* gene in a methylation-dependent manner. Both USF1 and USF2 are ubiquitous proteins characterized by highly conserved C-terminal basic helix-loop-helix and leucine zipper domains responsible for their dimerization and DNA binding activities [23], [26]. A number of genes have an E-box in their promoter, and the consensus sequence CANNTG, is known as the binding site for USFs to regulate transcription [27], [28]. For example, Mitf activates transcription through binding to the E-box element on the regulatory sequences of three germ genes (*dazl*, *dnd* and *vasa*) in medaka [29]. Although expressed ubiquitously, USFs appear to be involved in regulation of developmental and tissue-specific expression of other target genes such as surfactant protein A, steroidogenic factor 1, chipmunk hibernation-specific HP-27, transcription factor homeobox B4, carboxyl ester lipase and Prolyl-4-hydroxylase (I) [30], [31], [32], [33], [34], [35]. Our results show that USF is required for the specific expression of the *Miwi* gene. This USF regulation is methylation-dependent, which ensures developmental and cell type-specific expression of *Miwi* during meiosis of male germ cells.

The methylation of genomic DNA at CpG dinucleotides is a major epigenetic modification of the genome [36] and contributes to general transcriptional suppression, either by blocking the binding of transcription factors to their CpG-containing binding sites [31] or by recruiting methyl-CpG-binding proteins, which in turn recruit chromatin-remodeling machinery to induce the formation of a repressive chromatin structure [37]. Promoter methylation is associated with down-regulation of gene expression during spermatogenesis [38], [39], [40]. *Miwi* is a gene expressed specifically in the testis from midpachytene spermatocytes to round spermatids. To address how *Miwi* gene is regulated during spermatogenesis, we used bisulfite genomic sequencing to profile CpG methylation within the proximal promoter in cells where the gene is transcribed or silenced. We indeed observed an obvious correlation between *Miwi* gene expression and CpG methylation status in concert with differentiation of germ cell types. Importantly, we observed a key binding site for transcription factor USF, which plays a vital role in regulation of *Miwi* expression in a demethylation dependent manner. A selective demethylation and a consequent remethylation late in germ cell development have also been observed in several other testis-specific genes such as *ALF* and *Pgk2* [41], [42], [43]. Taken together, these data support that methylation modification is a key regulation mechanism for spermatogenesis.

In mammals, both DNA methylation and demethylation events occur during germ cell development and differentiation. Molecular mechanisms of regulating these processes remain largely unknown. There are five methyltransferases (DNMTs), DNMT1, DNMT2, DNMT3a, DNMT3b and DNMT3L [44], and three of them DNMT1, DNMT3a, and DNMT3b are involved in methylation *in vivo*. It has been proposed that DNMT3a and DNMT3b may contribute differentially to the establishment and/or maintenance of methylation patterns in male germ cells [45], [46]. Understanding of mammalian DNA demethylation represents a great challenge. There are probably two kinds of molecular mechanisms of DNA demethylation in mammals [47], passive demethylation occuring by blocking methylation of newly synthesized DNA during DNA replication, and active demethylation which is demethylation process is initiated by the same enzymes that establish the methylation, DNMT3A and DNMT3B [48], [49]. The question remains, as to how the demethylation of such an important E2 cis-element is regulated during spermatogenesis. Further study on the mechanism will provide new insight into mammalian spermatogenesis.

## Materials and Methods

All animal work has been conducted according to relevant national and international guidelines. Detailed Materials and Methods are described in Text S1.

### *In silico* sequence analysis

Transcription factor binding sites were predicted in the genomic DNA sequence of mouse *Miwi* using the TFsearch program (http://www.cbrc.jp/research/db/TFSEARCH.html) with a threshold score of 85 and Vertebrate Matrix. CpG islands in *Miwi* promoter were predicted by MethPrimer (http://www.urogene.org/methprimer/index1.html) with observed/expected ratio >0.6 and percent C+G>50%.

### Cell culture, transient transfection, and dual-luciferase reporter assay

GC-1 (a mouse germ-cell line at a stage between a type B spermatogonia and primary spermatocyte, has the capacity to differentiate into spermatids within the seminiferous tubules.) and COS7 (African Green Monkey SV40-transformed kidney fibroblast cell line) cells (for comparison, both germ-line and non germ-line cells were chosen) were maintained in high glucose Dulbecco's modified Eagle's medium supplemented with 10% fetal bovine serum, and plated in 48-well plates and transfected using 2 µl Lipofectamine 2000 (Invitrogen, Carlsbad, CA, USA) in each well. Plasmid DNAs were prepared in a dam- and dcm- E.coli strain (SCS110). Each deletion construct (pGL3-5M1, pGL3-5M2, pGL3-5M3, pGL3-5M4, pGL3-5M5, pGL3-5M6, pGL3-5M7 and pGL3-5M8) was transfected at 0.4 µg along with 10 ng/well pRT-TK (an internal control). For co-transfection luciferase assays, 0.2 µg of *Miwi* promoter construct pGL3-5M4 or mutated constructs, and 0.2 µg transcription factor expression vectors or corresponding dominant negative plasmid were co-transfected into cells. Empty vector pCX was added to equalize the final DNA content among each well. Luciferase activities were measured 24 h after transfection using the dual-luciferase reporter assay system (Promega, Madison, WI, USA) and a Modulus Single Tube Multimode Reader (Turner Biosystems, Sunnyvale, CA, USA) based on the manufacturer's protocol. Assays were performed in triplicates and expressed as means ± S.D.

### Isolation of spermatogenic cells by fluorescence-activated cell sorting (FACS)

Spermatogonia, pachytene spermatocytes, round spermatids, and Sertoli cells were isolated using the method described by Mays-Hoopes et al. [50]. Briefly, after the tunica albunigea was removed, the seminiferous tubules of adult testis of KunMing mice were treated with collagenase and washed to release the Leydig cells as well as interstitial cells. 0.25% trypsin treatment for 20 min at 37°C was followed by pipetting to release the cells. After fixed by the high citrate fixative method, the cells were ready for fluorescence-activated cell sorting.

For separation and collection of the EGFP-positive and negative cells from the testis of *Miwi*-*EGFP* transgenic mice, the seminiferous tubules were treated with trypsin and pipetted to release the cells. The cells were then analyzed on a MoFlo-XDP High-performance cell sorter (Beckman Coulter, Inc., Fullerton, CA, USA). Excitation of GFP was at 488 nm. Sorted cell populations were collected by centrifugation for 3 min at 3000 g and resuspended in PBS.

### Bisulfite–PCR methylation analysis

Sodium bisulfite treatment of genomic DNA was performed as follows. Briefly, 2 µg of genomic DNA was denatured with 2 M NaOH for 15 min, then treated with 3 M sodium bisulfate (pH 5.0) (Sigma-Aldrich, St.Louis, MO, USA) and 10 mM hydroquinone at 50°C for 18 h. DNA was purified using a Wizar DNA Clean-up kit (Sigma-Aldrich, St Louis, MO, USA), and NaOH was added to 0.3M. Bisulfite treated DNA, which was then precipitated with ethanol and resuspended in 20 µl sterile distilled water.

### Electrophoretic mobility shift assays (EMSA)

Oligonucleotides corresponding to the CCAAT and E boxes of the *Miwi* promoter were synthesized and annealed into double strands. Radiolabeled probes were generated by incubation of 250 ng annealed oligonucleotides with 20 µCi [γ-^32^P] dATP in the presence of T4 Polynucleotide Kinase (Promega, Madison, WI, USA) for 1 h at 37°C, and were subsequently separated from free nucleotide using G-50 column purification (Amersham Biosciences, Uppsala, Sweden). Testis nuclear extract was then incubated at room temperature for 30 min with a 100,000 dpm radiolabeled probe and 1 µg poly (dI-dC) in 10 mmol/l Tris-HCl, pH 7.5, 50 mmol/l NaCl, 1 mmol/l dithiothreitol, 1 mmol/l EDTA, and 5% glycerol. For supershift experiments, binding reactions were subsequently incubated with 5 µg of antibody for 30 min at room temperature. For competition experiments, the unlabeled competitor oligos (in 50-fold molar excess) were added together with the probe at the start of the incubation. Samples were resolved on 5% polyacrylamide gels in 0.5% TBE running buffer at 10 V/cm for 2 h. The dried gel was exposed to a phosphorimager cassette and scanned with typhoon 9200 instrument (GE-Healthcare, Amersham bioscience, Uppsala, Sweden).

### Chromatin immunoprecipitation (ChIP)

Mouse testis and livers were chopped into small pieces with a scalpel in cold phosphate-buffered saline (PBS) and cross-linked in 1% formaldehyde-PBS for 15 min with constant shaking. The tissue was rinsed in cold PBS and homogenized with a Dounce homogenizer in 1 ml cold cell lysis buffer (10 mM Tris-Cl, pH 8.0, 10 mM NaCl, 3 mM MgCl2, 0.5% NP-40) supplemented with protease inhibitors (Roche Diagnostics Ltd, Mannheim, Germany). Cells were incubated at 4°C for 5 min to allow the release of nuclei. Nuclei were sedimented by centrifugation at 13,000 g for 5 min. After lysis in the buffer (1% sodium dodecyl sulfate [SDS], 5 mM EDTA, 50 mM Tris-Cl, pH 8.1), sonication was performed with a Sonic Dismembrator model 100 sonicator (Fisher Scientific, Inc., Pittsburgh, PA, USA). After centrifugation, the supernatant chromatin was immunoprecipitated with no antibody (beads only), preimmuno IgG (IgG), anti-NF-Ya (Abcam Inc., Cambridge, CA, USA), anti-USF1 (Santa Cruz Biotech, CA, USA) or anti-USF2 (Santa Cruz), together with Protein G PLUS-Agarose (Santa Cruz) respectively. DNA isolated from the immunoprecipitated complex was amplified by PCR with primers flanking the CCAAT/E box binding sites or control primers. The PCR products were cloned into T-easy vector (Promega, Madison, WI, USA) and sequenced.

### Production of transgenic mice

A 303 bp fragment of mouse *Miwi* promoter ranging from −122 to +181 was amplified from genomic DNA of the KunMing mouse using primers designed with an EcoRI recognition site at the 5′ end of the forward primer and a BamHI recognition site on the 5′ end of the reverse primer. PCR fragments were subcloned into EcoRI/BamHI-digested pEGFP-1 vector. A 1.3 kb fragment digested with both HindIII and AflII was separated by agarose gel electrophoresis, dissolved in buffer of 5 mM Tris and 0.1 mM EDTA at 1 ng/µl. The purified DNA was microinjected into pronuclei of fertilized eggs from the KunMing mice on an inverted microscope (Leica DM IRB) (Leica Microscopy Systems Ltd, Wetzlar, Germany) equipped with a Leica manually operated micromanipulator (Leica) attached to a micro-injection system (Narishige, Tokyo, Japan). The injected eggs were transferred into the oviduct of day 1 pseudopregnant recipients. Transgenic animals were identified by PCR screening of genomic DNA isolated from tail with a pair of primers in the transgenic fragment and GFP checking of testis sections under fluorescent microscopy (Leica). Two independent transgenic lines have been analyzed in this study. Homozygotes were identified by crossing experiments and 100% presence of transgene in offsprings.

### Immunofluorescence and immunocytochemistry

Mouse testes were embedded in OCT medium (Tissue Tek, Miles, Elkhart, IN, USA) and cut into a series of 6 µm sections with a cryostat (Leica, Bensheim, Germany). To determine immunocytochemical localization, the sections were fixed with methanol for 20 min at −20°C and then permeabilized with 0.1% Triton X-100 in PBS for 30 min. Then sections were treated with 5% BSA for 20 min at room temperature and incubated with anti-Miwi (G82) (Cell Signaling Technology Inc., Danvers, MA, USA) or anti-GFP (D5.1) (Cell Signaling Technology). Then SABC and DAB were used for color visualization according to the manufacturer's instructions (Boster Company, China). For immunofluorescence analysis, after sections were fixed with methanol for 20 min at −20°C, permeabilized with 0.1% Triton X-100 in PBS for 30 min, and treated with 5% BSA for 20 min at room temperature, the sections were incubated at 4°C overnight in 1∶50 anti-Miwi (Cell Signaling Technology). After washing 3 times with PBS, 1∶100 dilution of the corresponding DylightTM 594-conjugated secondary antibody (Jackson ImmunoResearch Laboratories, Inc., West Grove, PA, USA) was applied at 37°C for 1 hour. The sections were washed in PBS for 5 times. After blocked again at room temperature for 20 min in 5% BSA, the sections were incubated at 4°C overnight in 1∶50 anti-GFP (Abcam). The corresponding FITC-conjugated secondary antibody (1∶100 dilution)(Proteintech Group, Chicago, IL, USA) were then incubated at 37°C for 1 hour. The nuclei were counterstained with Hoechst33258. Images were taken by confocal fluorescence microscopy (FV1000, Olympus, Tokyo, Japan).

## Supporting Information

Figure S1 Negative controls for immunofluorescence and immunocytochemical experiments. A, Immunofluorescence and confocal microscopy analysis of *Miwi*-*EGFP* transgenic mouse testis using goat serum or rabbit serum as a negative control (for Figure 6). B, Immunocytochemical analysis of wild type mouse testis using rabbit serum as a negative control (for Figure 7).
(TIF)

Text S1 Methods for making all DNA constructs and bisulfite–PCR methylation analysis are described. All primers and oligos are listed.
(DOC)
