# Supplementary figures and images for: DNA Demethylation and USF Regulate the Meiosis-Specific Expression of the Mouse Miwi
Source: PLoS Genet. 2012 May 17;8(5):e1002716. doi: 10.1371/journal.pgen.1002716 (PMC3355075; doi:10.1371/journal.pgen.1002716)

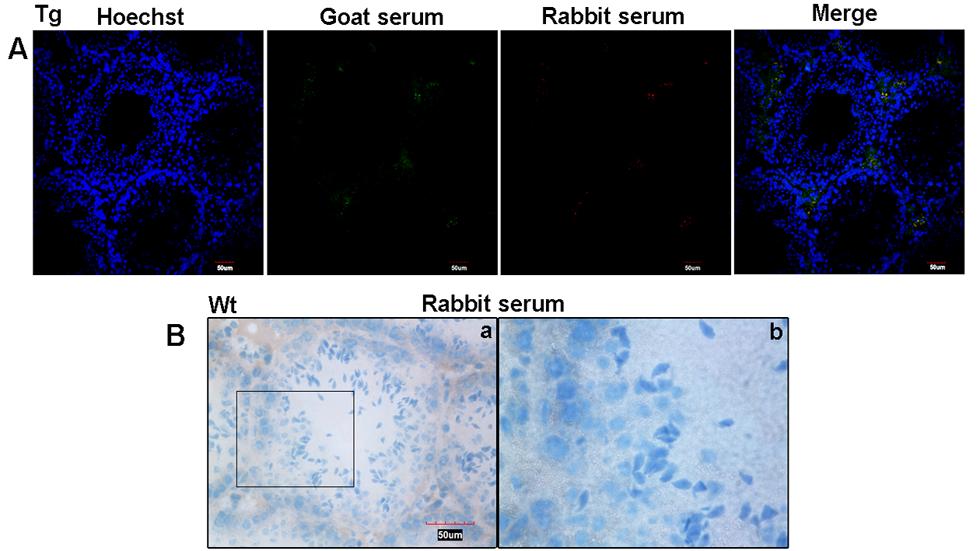

Supplement: Figure S1 — Negative controls for immunofluorescence and immunocytochemical experiments. A, Immunofluorescence and confocal microscopy analysis of Miwi-EGFP transgenic mouse testis using goat serum or rabbit serum as a negative control (for Figure 6). B, Immunocytochemical analysis of wild type mouse testis using rabbit serum as a negative control (for Figure 7). (TIF) [file pgen.1002716.s001.tif]
