# Supplementary material for: DNA Demethylation and USF Regulate the Meiosis-Specific Expression of the Mouse Miwi
Source: PLoS Genet. 2012 May 17;8(5):e1002716. doi: 10.1371/journal.pgen.1002716 (PMC3355075; doi:10.1371/journal.pgen.1002716)
Supplement: Text S1 — Methods for making all DNA constructs and bisulfite–PCR methylation analysis are described. All primers and oligos are listed. (DOC) [file pgen.1002716.s002.doc]

**Materials and Methods S1**

***Miwi* promoter deletion mutants, site-directed mutagenesis for the CCAAT and E boxes and construction of expression plasmids**

Eight *Miwi* promoter deletion mutants were constructed by PCR cloning with the primers: forward primers: pGL3-5M1, 5′CCGACGCGTTCATCTGCAGTGGGTTTTGGCAC3′; pGL3-5M2, 5′CCGACGCGTGCCACCATTGAGTGAGGAGGATT3′; pGL3-5M3, 5′CCGACGCGTCCAAGAAGAATGGGGCTTGG3′; pGL3-5M4, 5′CCGACGCGTCACAAA ACCGCCACCCACGTG3′; pGL3-5M5, 5′CCGACGCGTACGTGGCAGCCAATCAGGGC3′; pGL3-5M6, 5′CCGACGCGTCAGGGCCCAGCACGTGTCCA3′; pGL3-5M7, 5′ CCGACGCGTTCCACCTCGCCGTTAACCCA3′; pGL3-5M8, 5′CCGACGCGTAGGGATCTGGCAGTTGGGGCTGT3′, and a common reverse primer 5′CCCAAGCTTCCTGATCGTCGTGGTCCAGC3′. PCR products were double-digested with MluI and HindIII, and cloned into the MluI/HindIII sites of the pGL3-basic vector (Promega, Madison, WI, USA). All the DNA constructs were confirmed by DNA sequencing.

Site-directed mutagenesis for the CCAAT and E boxes was carried out using primers as follows:

pGL3-5M4-Cmut for CCAAT box mutant, 5′CCACCCACGTGGCAGTTCCTCAGGGCCCAGCACG3′ and 5′CGTGCTGGGCCCTGAGGAACTGCCACGTGGGTGG3′; pGL3-5M4-E1mut for E1 box mutant, 5′ ACAAAACCGCCACCTCTAGTGCAGCCAATCAGGG3′ and 5′CCCTGATTGGCTGCACTAGAGGTGGCGGTTTTGT3′; pGL3-5M4-E2mut for E2 box mutant, 5′CAATCAGGGCCCAGTCTAGTTCCACCTCGCCGTT3′ and 5′ AACGGCGAGGTGGAACTAGACTGGGCCCTGATTG3′. pGL3-5M4-E1/2mut mutant for both E boxes using pGL3-5M4-E1mut as template, 5′ CAATCAGGGCCCAGTCTAGTTCCACCTCGCCGTT3′ and 5′AACGGCGAGGTGGAACTAGACTGGGCCCTGATTG3′. All constructs were sequenced.

For construction of expression plasmids, the full-length cDNAs of mouse wild-type NF-Ya, NF-Yb, NF-Yc, USF1 and USF2 were PCR-amplified from mouse testis cDNA and cloned into EcoRI site of the pCX-EGFP vector by replacing the EGFP fragment to generate expression plasmids (pCX-NFYa, pCX-NFYb, pCX-NFYc, pCX-USF1 and pCX-USF2) using primers: NF-Ya, 5′CGTGAATTCGCCATGGAGCAGTATA3′ and 5′CTCGAATTCTTAGGAAACTCGGATGA3′; NF-Yb, 5′ATAGAATTCATCATGACAATGGACGG3′ and 5′ TCCGAATTCTCATGAAAACTGAATTTG3′; NF-Yc, 5′GCCGAATTCAAAATGTCCACAGAAG3′ and 5′CTCGAATTCTCAGTCTCCAGTCACCT3′; USF1, 5′CCTGAATTCGAGweATGAAGGGGCAGC3′ and 5′TCCGAATTCTTAGTTGCTGTCATTCTTG3′; USF2, 5′CCGAATTCACCATGGACATGCTGGA3′ and 5′CGGGAATTCTCACTGCCGGGTACTC3′. pCX-EGFP vector deleted the EGFP cDNA was used as a control plasmid (pCX). The mouse dominant negative NF-Ya and USF plasmids (NF-YA29 and A-USF) were generous gifts from Dr. Roberto Mantovani (University of Milano) and Dr. Charles Vinson (National Institutes of Health).

**Bisulfite–PCR methylation analysis.**

After purified the bisulfite treated DNA, two rounds of PCR amplification were performed using primers: for CpG island 1, CG1-5,5′ TATTTGTAGTGGGTTTTGGTATAGG3′, CG1-3, 5′ CTCCTCACTCAATAATAACCCTTC3′ and a nest primer CG1-3nest, 5′ TCCATCTCTAACTAACCTTAAAAATC3′; for CpG island 2/3, CG23-5, 5′ GTTGGAAGGGTTATTATTGAGTGAG3′, CG23-3, 5′ CGAAATCAAAACACAAATAAACACA3′ and a nest primer CG23-3nest, 5′ CACAACTTAATTCCAAACCCTAATC3′. The PCR products were cloned into T-easy vector (Promega, Madison, WI, USA) and sequenced.

For Bisulfite–PCR analysis of the *Miwi* promoter driven transgene in the transgenic mouse, the bisulfite treated DNA from EGFP-positive and EGFP-negative cells were purified, two rounds of PCR amplification were performed using primers: Tg-BSP-5, 5′ GAATTTTATAAAATCGTTATTTA3′, Tg-BSP-3, 5′ACCAAAATAAACACCACCCC3′, and a nest primer Tg-BSP-3 nest, 5′CCTTACTCACCATAATAAC 3′. The PCR products were cloned into T-easy vector (Promega, Madison, WI, USA) and sequenced.

**Oligos for electrophoretic mobility shift assays**

Their sequences are as follows: oligo1, 5′GGCAGCCAATCAGGGCCCAGCAC3′ and 5′GTGCTGGGCCCTGATTGGCTGCC3′; Oligo1-ccaat mut, 5′GGCAGTTCCTCAGGGCCCAGCAC3′ and 5′GTGCTGGGCCCTGAGGAACTGCC3′; Oligo2, 5′GGCAGCCAATCAGGGCCCAGCACGTGTCCACC3′ and, 5′GGTGGACACGTGCTGGGCCCTGATTGGCTGCC3′; Oligo2-ccaat mut, 5′GGCAGTTCCTCAGGGCCCAGCACGTGTCCACC3′ and 5′GGTGGACACGTGCTGGGCCCTGAGGAACTGCC3′; Oligo2-E2 mut, 5′GGCAGCCAATCAGGGCCCAGTCTAGTTCCACC3′ and 5′GGTGGAACTAGACTGGGCCCTGATTGGCTGCC3′; Oligo2-ccaat/E2 mut, 5′GGCAGTTCCTCAGGGCCCAGTCTAGTTCCACC3′ and 5′GGTGGAACTAGACTGGGCCCTGAGGAACTGCC3′; Oligo3, 5′CAGGGCCCAGCACGTGTCCACC3′ and 5′GGTGGACACGTGCTGGGCCCTG3′; Oligo3-E2 mut, 5′CAGGGCCCAGTCTAGTTCCACC3′ and 5′GGTGGAACTAGACTGGGCCCTG3′.

For the methylated oligo3, Single stranded oligonucleotide probes were duplexed, methylated with M.Sss1 *in vitro*, then labeled with γ-^32^P-ATP.

**Primers for ChIP**

The ChIP primer sequences are: 5′GCCACCATTGAGTGAGGA3′ and 5′ACAGCCCCAACTGCCAG3′. The amplified PCR fragments were analyzed on 2% agarose gel. A region of 215 bp flanking exon 6 of the *Miwi* was amplified as a control with primers: 5′GCTCCTCTGGCATCATTGTG3′ and 5′CTTGTGCTGTAGTCTCTTAGGTAAA3′.

**Primers for screening transgenic mice**

Primers for amplification of *Miwi* promoter: forward primer: 5′CCGGAATTCCACAAAACCGCCACCCACGTG3′ and reverse primer: 5′GTTGGATCCCCTGATCGTCGTGGTCCAGC3′. PCR screening for transgenic mice with primer pairs: 5'TCTGGCAGTTGGGGCTGTTAGC3' and 5' TTGATGCCGTTCTTCTGCTTGTC3'.
